# Construction of the first high-density SNP genetic map and identification of QTLs for the natural rubber content in *Taraxacum kok-saghyz* Rodin

**DOI:** 10.1186/s12864-022-09105-3

**Published:** 2023-01-10

**Authors:** Yushuang Yang, Bi Qin, Qiuhui Chen, Qiuhai Nie, Jichuan Zhang, Liqun Zhang, Shizhong Liu

**Affiliations:** 1 Rubber Research Institute, Chinese Academy of Tropical Agricultural Science, 571101 Haikou, China; 2 Beijing Linglong Dandelion Technology and Development Ltd, 101102 Beijing, China; 3 College of Materials and Engineering, Beijing University of Chemical Technology, 100029 Beijing, China

**Keywords:** *Taraxacum kok-saghyz* Rodin, Natural rubber content, Genetic map, Single nucleotide polymorphism, Quantitative trait locus, Whole-genome resequencing

## Abstract

**Background:**

*Taraxacum kok-saghyz* Rodin (TKS) is a promising commercial alternative natural rubber (NR) yielding plant. Cultivating TKS with a high NR content is an important breeding target, and developing molecular markers related to NR content can effectively accelerate the breeding process of TKS.

**Results:**

To construct a high-density SNP genetic map and uncover genomic regions related to the NR content in TKS, an F_1_ mapping population of TKS was constructed by crossing two parents (l66 and X51) with significant differences in NR contents. The NR content of the F_1_ plants ranged from 0.30 to 15.14% and was distributed normally with a coefficient of variation of 47.61%, indicating quantitative trait inheritance. Then, employing whole-genome resequencing (WGR), a TKS genetic linkage map of 12,680 bin markers comprising 322,439 SNPs was generated. Based on the genetic map and NR content of the F_1_ population, six quantitative trait loci (QTLs) for NR content with LOD > 4.0 were identified on LG01/Chr01 and LG06/Chr06. Of them, the 2.17 Mb genomic region between qHRC-C6-1 and qHRC-C6-2 on ChrA06, with 65.62% PVE in total, was the major QTL region. In addition, the six QTLs have significant additive genetic effects on NR content and could be used to develop markers for marker-assisted selection (MAS) in TKS with a high NR content.

**Conclusion:**

This work constructed the first high-density TKS genetic map and identified the QTLs and genomic regions controlling the NR content, which provides useful information for fine mapping, map-based cloning, and MAS in TKS.

**Supplementary Information:**

The online version contains supplementary material available at 10.1186/s12864-022-09105-3.

## Introduction

*Taraxacum koksaghyz* Rodin (TKS) is a rosette-shaped perennial herb of Compositae with a genome of 1.07 Gb [1, 2] that originates in the Tian Shan mountains of China and Kazakhstan [3]. Its roots can produce and store NR in high quantities (reaching 20% dry weight) and with a high molecular weight (2180 KD on average), comparable to that of the rubber tree [4]. Additionally, TKS is adapted to grow in temperate regions and can be cultivated and harvested mechanically and annually. Therefore, TKS is a promising commercial alternative NR-yielding plant in temperate regions [5]. Although obvious progress has been achieved in breeding for cultivated TKS as an industrial crop in recent decades [6], NR production in TKS still does not meet the requirements for large-scale commercial planting and production, and TKS breeding efficiency is still low due to its high heterozygosity, poor vigor and competitiveness in the field and self-incompatibility inbreeding depression [7].

NR content is one of the most important traits in TKS and urgently needs to be improved. To date, a total of 90 functional genes controlling the NR synthesis pathway in TKS have been identified, including 32 genes in the mevalonic acid (MVA) pathway, 19 genes in the 2-C-methyl-D-erythritol-4-phosphate (MEP) pathway, 17 genes in NR synthesis initiation and 22 genes in NR chain extension [1]. Additionally, many transcription factors (TFs) regulating NR biosynthesis have been identified [8–11]. However, the failure of many overexpressed NR biosynthesis genes to effectively improve the NR production capacity of TKS may be due to inadequate knowledge of the molecular mechanisms of NR biosynthesis [12–14]. Therefore, the NR content, as a complex trait controlled by multiple pathways and genes, needs further exploration.

Molecular markers play a pivotal role in efficiently developing and improving crop germplasm. They are practical and essential for high-throughput genotype identification in the seedling stage [15], genetic and evolutionary analysis, genetic map construction and QTL mapping for important economic traits [16–18]. To accelerate the TKS breeding process through MAS, the first TKS genetic map was constructed in 2016 [19]. It consisted of eight linkage groups (LGs) and included 1448 AFLP, 6 COS, 1 SSR and 63 EST-SSR markers. Then, hundreds of SSR markers were developed for diversity analysis in TKS [7, 20, 21]. With the emergence of next-generation sequencing (NGS), SNP markers have become widely favored because of their high abundance, even distribution and compatibility [22]. In 2017, a total of 21,036 SNPs were developed from the first TKS root transcriptome [23]. Among them, 50 SNPs were obtained from 39 transcripts related to rubber biosynthesis, and 117 SNPs were found in 36 differentially expressed gene sequences. In 2021, a total of 524,812 SNPs were obtained by genotyping-by-sequencing (GBS) and were used for population structure, genetic diversity, and evolutionary analysis of 58 wild TKS plants collected from four different regions [24].

Numerous SNP markers obtained by high-throughput sequencing could be used for constructing high-density genetic maps and QTL mapping for important traits [18, 25], and this approach has proven effective in many major crops [17, 26–28]. Recently, through further splicing and optimizing the TKS genome constructed in 2018 [2], a TKS genome sketch at the chromosome level was published in 2021, which provided a solid foundation for high-density SNP genetic map construction, QTL fine mapping and MAS for TKS breeding [1].

To construct a high-density TKS genetic map and identify QTLs for the NR content, an F_1_ mapping population of TKS derived from two parents (166 and X51) with significant differences in their NR content was constructed. Based on this F_1_ population, the first high-density TKS genetic map was constructed based on the bin markers derived from WGR. Subsequently, QTLs for the NR content were mapped and analyzed. The results lay a solid foundation for verifying candidate genes, gene cloning and MAS in TKS.

## Results

### Phenotypic analysis of the NR content in TKS

**Fig. 1:**
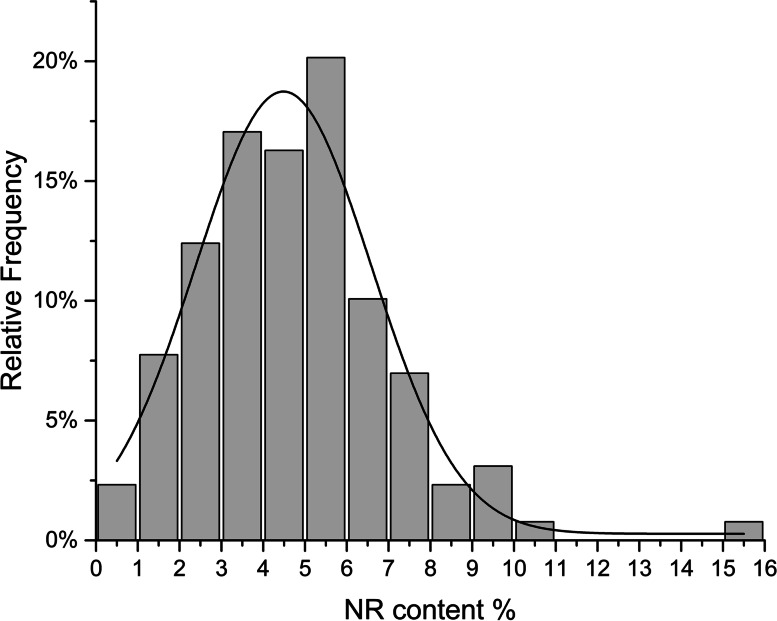
Frequency distribution for the NR content in the F_1_ segregating population

The F_1_ mapping population composed of 127 offspring was constructed by crossing the two TKS lines (166 and X51) that differ significantly in NR content, and their NR content was determined. As shown in Table 1; Fig. 1, the NR content of the F_1_ population ranged from 0.30 to 15.14% with a variation coefficient of 47.61%, indicating that the NR content of the F_1_ population had larger phenotypic separation. Transgressive segregation in both directions was observed. Among them, the NR content of five superparent offspring was higher than that of the high NR content parent X51 (9.21%), which laid a foundation for subsequent genetic analysis and high NR content breeding. According to the skewness and kurtosis in Table 1, the NR content of the F_1_ generation shows an approximately normal distribution, indicating that NR content is a quantitative trait controlled by multiple genes.

**Table 1:** Statistics of the natural rubber content distribution in the segregating F_1_ population

Material	Mean	Minimum %	Maximum %	CV %	Skewness		Kurtosis
P1(166)	2.09%						
P2(X51)	9.21%						
F_1_ progenies	4.75%	0.30%	15.14%	47.61%		0.94	2.59

### Whole genome resequencing (WGR) data and SNP marker analysis

The WGR of the 127 F_1_ progenies and two parental lines generated 1356.13 Gbp high-quality reads in total after quality trimming. The average Q30 ratio reached 93.97%, and the average GC content was approximately 38.29% (Table S1). The data of the SNP mutation distribution and the high-quality reading assembly scaffold coverage indicated that the WGR data were sufficiently random (Table S1, Figs. S1 and S2). The average mapping rate was 98.55%, and the average coverage depths for the two parent lines and their F_1_ progenies were 14-fold and 7.43-fold, respectively (Table S1). Finally, a total of 8,776,512 SNPs were discovered by SNP identification and genotyping. Among the SNPs, 322,439 were obtained by comparing the differences between the parents and F_1_ progenies and were integrated into 12,680 bins with lengths ≥ 1000 bp (Table 2 and Table S2).

**Table 2:** Distribution of genetic markers on the TKS genetic map

LG ID	Chromosome	Bin marker number	Physical distance bp	Average physical distance bp	Total genetic distance cM	Average genetic distance cM	Max gap (cM)	Gaps < 5 cM %
LG00	ChrA0	247	2,380,276	9636.74	515.10	2.09	25.16	94.31
LG01	ChrA1	1909	14,238,719	7458.73	5236.79	2.74	30.01	88.63
LG02	ChrA2	1908	14,755,488	7733.48	5231.68	2.74	29.25	87.52
LG03	ChrA3	1838	14,292,267	7775.99	5024.71	2.73	27.31	87.75
LG04	ChrA4	1461	11,737,559	8033.92	3370.78	2.31	29.23	90.68
LG05	ChrA5	1311	9,906,771	7556.65	3949.08	3.01	30.00	89.54
LG06	ChrA6	1509	10,352,151	6860.27	3949.08	2.62	26.39	89.85
LG07	ChrA7	1343	9,633,799	7173.34	3750.28	2.79	31.16	86.96
LG08	ChrA8	1154	8,582,201	7436.92	3193.25	2.77	29.67	87.51
Total		12,680	95,879,231		34,220.75			

### Construction of a high-density SNP genetic map

**Fig. 2:**
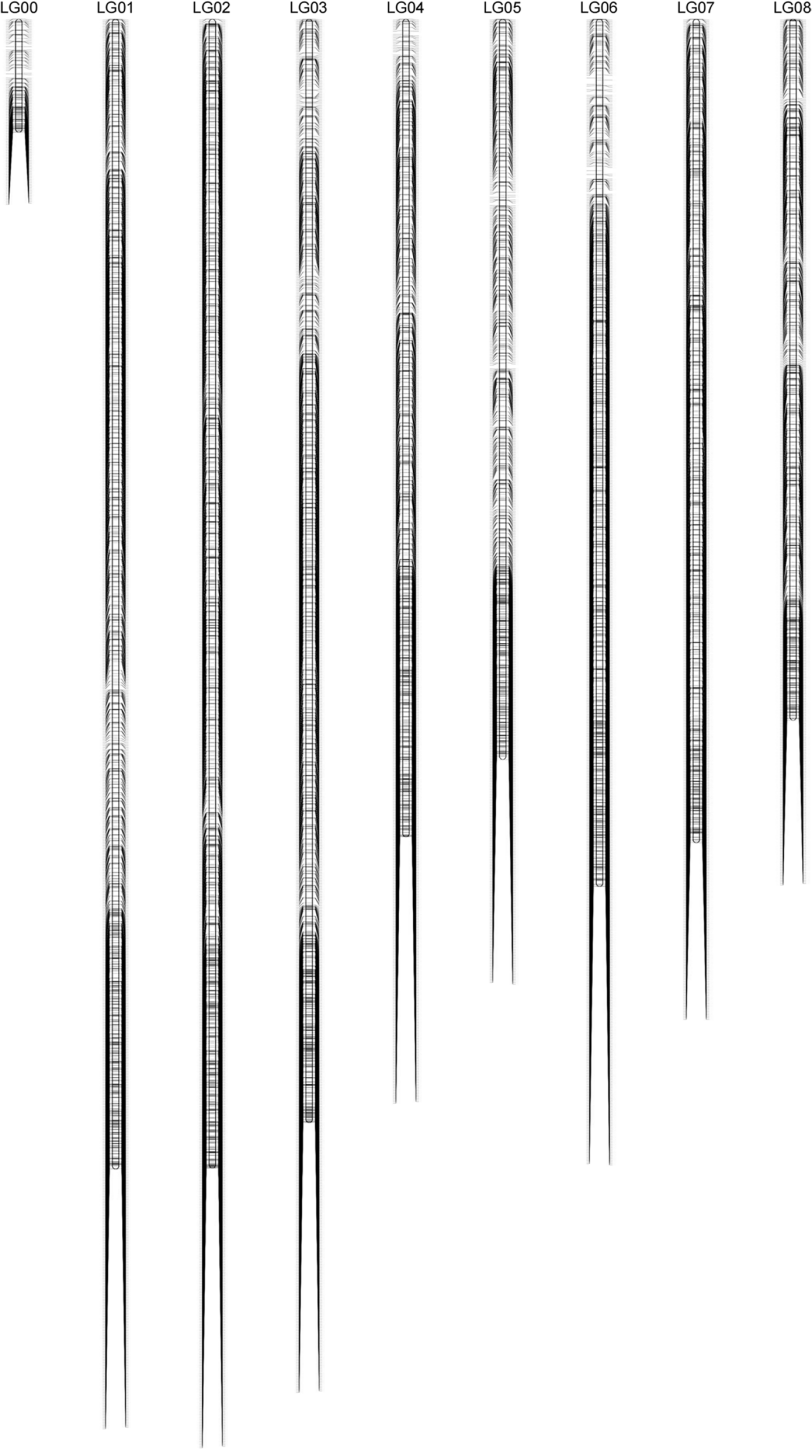
TKS genetic map constructed by bin markers

The final genetic map consisting of 9 LGs was constructed based on 12,680 bins containing 322,439 SNPs (Fig. 2). LG00 is the bin marker assembled on ChrA0, which is the sequence assembly that is not integrated into a specific chromosome.

The genetic length for the TKS genetic map was 34,220.75 cM in total, with an average genetic distance of 2.70 cM (Table 2). The proportion of the interval between adjacent bin markers less than 5 cM on average was approximately 89.19%, indicating that the bin markers were well distributed on the TKS genetic map. Additionally, the largest gap was 31.16 cM, which was located on LG07, and LG01 was the largest LG, covering 5236.79 cM and containing 1909 bin markers (Table 2).

**Fig. 3:**
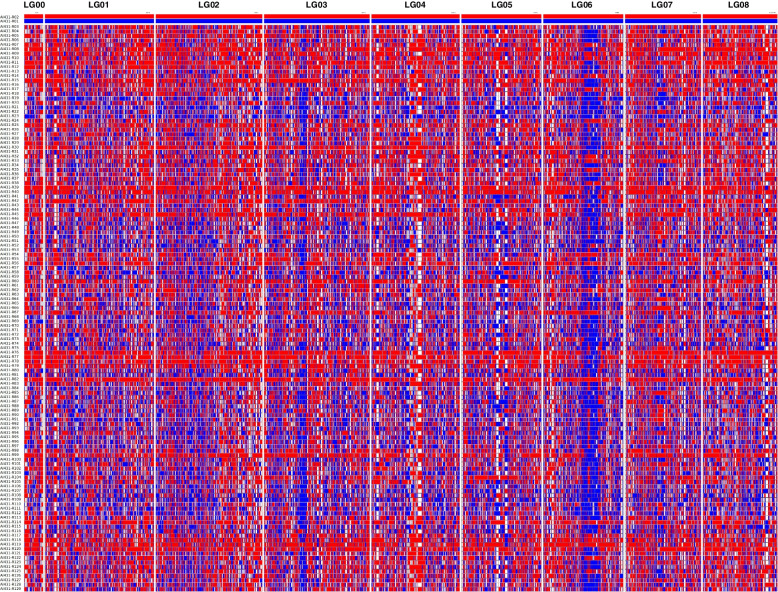
Haplotype map of the TKS genetic map. The x-axis represents the markers, and the y-axis represents the two parents and 127 F_1_ progenies. Blue represents parent line 166 (recessive homozygous genotype), red represents parent line X51 (dominant homozygous genotype), and white indicates the dominant heterozygous genotype

Haplotype maps were constructed for each of the 127 F_1_ progenies and two parental lines to evaluate the quality of the TKS genetic map. As illustrated in Fig. 3, almost all of the bin markers and the recombination blocks were clearly defined.

### Identification of QTLs related to the NR content in TKS

**Fig. 4:**
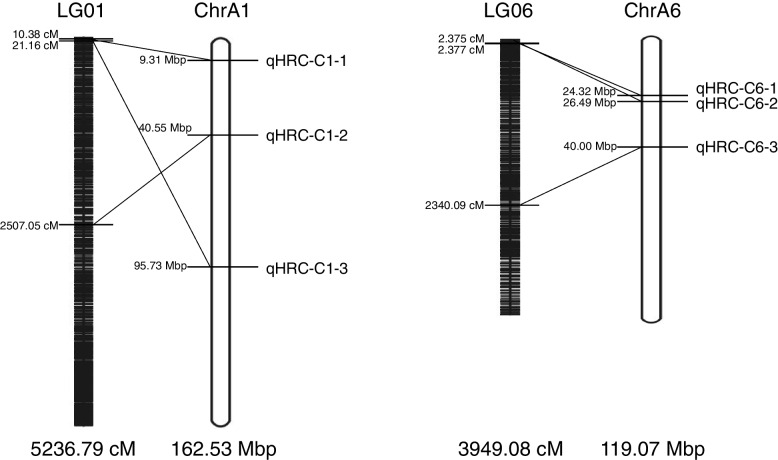
Location of the QTLs related to the NR content

As shown in Table 3; Fig. 4, six QTLs related to the NR content (LOD values ≥ 4.0) were identified and mapped on the corresponding linkage map. Among them, three QTLs (qHRC-C1-1, qHRC-C1-1 and qHRC-C1-2) were located on LG01/ChrA1, and the other three QTLs (qHRC-C6-1, qHRC-C6-2 and qHRC-C6-3) were located on LG06/ChrA6. On LG01/ChrA01, the QTL qHRC-C1-1 was located at 10.38 cM/9.31 Mbp, with a phenotypic variance value (PVE) of 17.89%, qHRC-C1-2 was located at 2507.05 cM/40.55 Mbp, with a PVE value of 12.59%, and qHRC-C1-3 was located at 21.16 cM/95.73 Mbp, with a PVE value of 19.44%. On LG06/ChrA6, the QTL qHRC-C6-1 was located at 2.377 cM/24.32 Mbp, with a PVE value of 24.45%; qHRC-C6-2 was located at 2.375 cM/26.49 bp, with a highest LOD value of 6.27 and a highest PVE value of 23.82%; and the QTL qHRC-C6-3 was located at 2340.09 cM/46.00 Mbp, with a PVE value of 17.35%.

The QTLs qHRC-C6-1 and qHRC-C6-2 with the 2 highest LOD values were located close to each other on LG06/ChrA06 (Fig. 4). The region between them on ChrA6 explained 48.27% of the PVE in total, indicating that it may be a major locus affecting the NR content in TKS. A total of 122 genes were predicted in the 2.17 Mb region between qHRC-C6-1 and qHRC-C6-2 (Table 3 and Table S4) on ChrA06. Among them, 35 were annotated in the nonredundant (NR) database (Table S4), and 87 genes had unknown functions in this region. The KEGG pathway enrichment analysis of these 35 genes showed that their functions included metabolism and genetic information processing (Table S5).

**Table 3:** Information on NR content-related QTLs in TKS

QTL ID	Bin marker ID	Physical position			Genetic position		LOD	PVE %
		Chr	Start	End	LG	Position		
qHRC-C1-1	ChrA1_9314111_9315229	ChrA1	9,314,111	9,315,229	LG01	10.38	5.15	17.89
qHRC-C1-2	ChrA1_40551944_40557463	ChrA1	40,551,944	40,557,463	LG01	2507.05	4.02	12.59
qHRC-C1-3	ChrA1_95729695_95742485	ChrA1	95,729,695	95,742,485	LG01	21.16	5.71	19.44
qHRC-C6-1	ChrA6_24318536_24322915	ChrA6	24,318,536	24,322,915	LG06	2.377	6.27	24.45
qHRC-C6-2	ChrA6_26486808_26487892	ChrA6	26,486,808	26,487,892	LG06	2.375	6.17	23.82
qHRC-C6-3	ChrA6_46000628_46002048	ChrA6	46,000,628	46,002,048	LG06	2340.09	5.11	17.35

### Genetic effects of QTLs related to the NR content

**Fig. 5:**
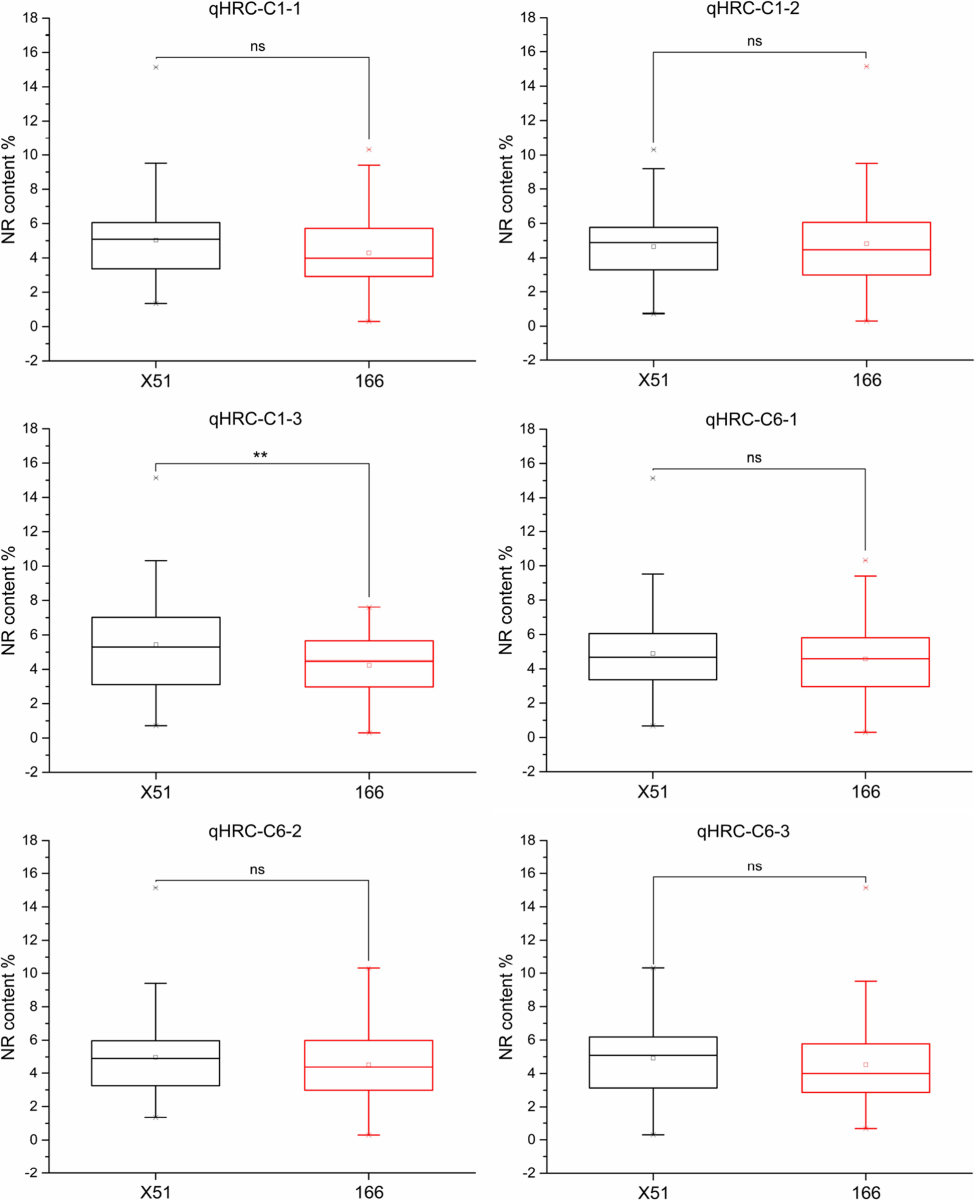
The genetic effects of QTLs on the NR content in the F_1_ population

Based on the genotype of linked markers, the genetic effects of the six major QTLs on the NR content were analyzed (Table S6, Table S7 and Fig. 5). Among them, qHRC-C1-1, qHRC-C1-3, qHRC-C6-1, qHRC-C6-2 and qHRC-C6-3 with X51 (High rubber content, HRC) alleles had positive effects (Fig. 5), but only qHRC-C1-3’s effect reached a significant level (*P* < 0.01). The NR content of lines with the X51(HRC) allele at qHRC-C1-1, qHRC-C1-3, qHRC-C6-1, qHRC-C6-2 and qHRC-C6-3 increased by 17.48%, 28.61%, 6.99%, 9.69% and 5.42%, respectively, relative to that in lines with the 166 (Low rubber content, LRC) allele. In contrast, qHRC-C1-2 with X51 (HRC) alleles had negative effects on the NR content, which decreased by 3.52% relative to that in lines with 166 (LRC) alleles (Fig. 5).

**Fig. 6:**
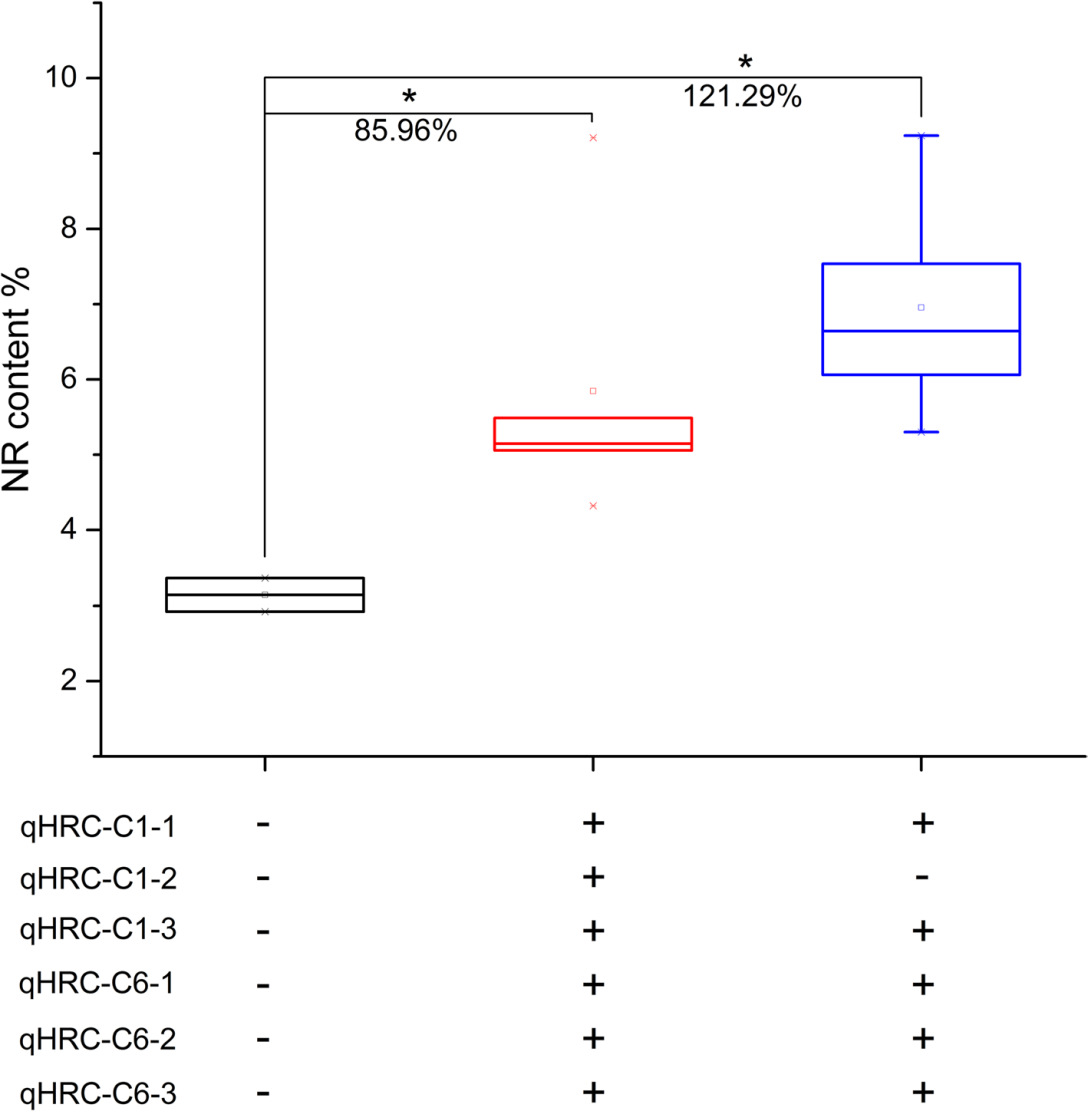
The additive effects of QTLs on the NR content in the F_1_ population. Note: + represents the genotype of parent X51; - represents the genotype of parent 166

The additive effects of the QTLs on the NR content were identified (Fig. 6 and Table S6). Different combinations of six QTLs affected the NR content of the F_1_ population to different degrees. Among them, compared to the lines with the L66 alleles at six QTLs, lines with X51 alleles at the six QTLs significantly increased the NR content by 85.96%, and lines with 166 alleles at qHRC-C1-2 and X51 alleles at the other five QTLs significantly increased the NR content by 121.29%, which was the QTL allele combination with the most significant additive effect.

## Discussion

### Construction of the first high-density TKS genetic map

Genetic mapping is essential for QTL mapping, map-based cloning, and MAS [29] and has been widely used in many major crops [17, 27, 28, 30]. In the past decade, although a great quantity of TKS molecular markers (SSRs and SNPs) have been developed, they have mostly been used in research on the genetic diversity and population structure of TKS [7, 20, 21, 23, 24]. In 2016, the first TKS genetic map consisting of eight LGs was constructed, which included 1448 AFLPs, 6 COSs, 1 SSR and 63 EST-SSRs [19]. However, the limited marker quantity and TKS genomic information available hindered the construction of high-density TKS genetic linkage maps. With the advent of NGS technology and TKS genome data [1], it is possible to construct a high-density genetic map for TKS. Due to the high genomic heterozygosity and self-incompatibility of TKS, the F_1_ population was generated for TKS genetic map construction, which has been successfully applied to many economic plants crops, such as rubber tree and loquat [31, 32]. With WGR data (7.43× coverage), we were able to genotype an F_1_ population in a genome-wide manner and construct a high-density TKS genetic map including 9 LGs (Fig. 2). It consisted of 12,680 bin markers representing 322,439 SNP markers that were well distributed on the genome (Table S1, Figs. S1 and S2). The genetic length for the TKS genetic map was 34,220.75 cM in total, with an average genetic distance of 2.70 cM. In contrast to the first genetic map reported in 2016 [19], the SNP identification and genetic map construction in this study were based on the TKS genome at the chromosome level [1], which improves the accuracy of SNP and bin marker positioning and arrangement. Moreover, the WGR-based genotyping method used in this study could be used to acquire more molecular markers (12,680 bin markers representing 322,439 SNPs) than previous marker-based genotyping methods (1448 AFLPs, 6 COSs, 1 SSR and 63 EST-SSRs) for constructing a genetic map, as has been proven in many crops [16, 29, 33]. In addition, there are many gaps exceeding 20 cM on the genetic map, and we further analyzed genotype data and recombinant events, and found fewer recombinant events in these genomic regions. It may be because the two parent lines of the F_1_ population have a consistent genotype in these specific chromosome segments and recombination cannot be detected, or there are some conservative chromosome regions, and no individual recombination occurs. However, the results above further confirmed the high density and high precision of the TKS genetic map.

### Identification of QTLs for the NR content and genetic effect analysis

The NR content is one of the most important traits in TKS. A larger number of studies have shown that the NR content should be a complex quantitative trait controlled by multiple genes and pathways [1, 12, 34]. In this study, the NR content phenotype of the F_1_ mapping population with 127 progenies ranged from 0.30 to 15.14% and was approximately normal distribution, further confirming that the NR content is a quantitative trait. Based on the preliminary QTL analysis results in this work, the LOD threshold was set at 4.0 to improve the reliability of QTL location and remove the interference of minor QTLs. Finally, a total of six QTLs were identified and mapped on the linkage map (Fig. 4; Table 3). Among them, qHRC-C6-1 and qHRC-C6-2 located on LG06/ChrA6 had the 2 highest LOD and PVE values, and the region among them was considered the major QTL region.

Pyramiding elite alleles has been proven to be a very effective method for crop trait improvement [33, 35]. Our results showed that qHRC-C1-2 with 166 (LRC) alleles and qHRC-C1-1, qHRC-C1-3, qHRC-C6-1, qHRC-C6-2 and qHRC-C6-3 with X51 (HRC) alleles had positive effects on the NR content (Fig. 5 and Table S6). In addition, different combinations of the six QTLs had additive effects on the NR content in the F_1_ population. Among them, compared to the lines with L66 alleles at six QTLs, lines with 166 alleles at qHRC-C1-2 together with X51 alleles at the other five QTLs, which was the QTL allele combination with the most significant additive effect, showed a significantly increased NR content by 121.29% (Fig. 6 and Table S6). The results above suggested that the genetic effects of the six QTLs related to the NR content were stackable with potential applications in marker development and MAS breeding in TKS. However, as a complex quantitative trait, the genetic expression of NR content is easily affected by environmental conditions, and the additive effect of the NR content of TKS plants with the same or different haplotypes in different years and environmental conditions may have obvious differences, which requires more experiments for verification.

### Candidate genes in the QTL regions related to the NR content in TKS

To explore candidate genes related to NR content in the six QTL regions, the gene information located in or near the six QTL regions was retrieved and analyzed according to the TKS genomic sequence and gene prediction information. Five of these QTLs (qHRC-C1-1, qHRC-C1-3, qHRC-C6-1, qHRC-C6-2 and qHRC-C6-3) are located in intergenic regions, only qHRC-C1-3 was located on a gene (TkA01G383590) encoding a putative FBD-associated F-box protein, which was identified to play a role in plant resistance to stress [36]. Surprisingly, no known key genes involved in NR synthesis were located near the six QTL regions, such as rubber cis-prenyltransferase (CPT) [13], cis-prenyltransferase-like (CPTL) [37], rubber elongation factor (REF) [38] and small rubber particle protein (SRPP) [39]. The same phenomenon was also found in the QTL mapping analysis of rubber latex yield in rubber tree [31]. Recently, a comparative root transcriptome analysis between the LRC and HRC lines in TKS indicated that there was substrate competition between NR and other metabolites, such as sesquiterpene, triterpenoid, diarylheptanoid, gingerol, flavonoid [34], which affected NR synthesis. Therefore, there may be some unknown NR synthesis-related genes located in these QTL regions, which need to be further explored.

The QTLs qHRC-C6-1 and qHRC-C6-2 with the 2 highest LOD values were located close to each other on LG06/ChrA6. The 0.002 cM/2.17 Mbp region between qHRC-C6-1 and qHRC-C6-2. (Table 3 and Table S4) on LG06/ChrA6 explained 48.27% of the phenotypic variance and was identified as the major region for the NR content trait. In this region, 122 genes were predicted, 35 were annotated in the nonredundant (NR) database (Table S4) and 87 genes with unknown functions in this region. Among them, one gene encoding 1-deoxy-d-xylulose-5-phosphate synthase (DXS) (TkA06G097470) was identified, with a physical position between 25,960,329 bp and 25,964,468 bp. It catalyzes the first step of the MEP pathway, which produces isopentenyl pyrophosphate (IPP) as the substrate for NR synthesis [40, 41]. One gene encoding geranylgeranyl diphosphate synthase 5 (GGPS5) (TkA06G097970) was identified, with a physical position between 26,084,844 bp and 26,085,707 bp. GGPS, which is involved in the initiator synthesis of NR, is a significant branching enzyme in terpenoid biosynthesis and functions in many agronomic traits (root biomass, flowering and seed yield, etc.) [42]. Moreover, the comparison results of the GGPS genes between TKS and TM with significant differences in NR contents showed that GGPS5 only existed in TKS and had a medium expression in latex, indicating a positive function for NR synthesis in TKS [1]. Additionally, six genes (TkA06G095920; TkA06G096310; TkA06G096400; TkA06G096500; TkA06G097850; TkA06G097930) (Table S4) located in this region are involved in the sesquiterpenoid and triterpenoid biosynthesis pathway, which competes with NR synthesis in the use of a common substrate (IPP) [34]. However, there is no direct evidence that these genes regulate the NR content, and further studies are needed for functional validation and fine mapping of the genes in these regions. The first high-density TKS genetic map and mapping results for the NR content will provide valuable information for candidate gene identification, gene cloning, and MAS breeding in TKS.

## Conclusion

In this work, the first high-density TKS genetic map was constructed based on WGR technology. It consists of 12,680 bin markers representing 322,439 SNPs that were well distributed on the TKS genome. The total genetic length was 34,220.75 cM, with an average distance of 2.70 cM. Then, a total of six QTLs related to the NR content were identified and had significant additive genetic effects on the NR content, which could be used to develop markers for MAS in TKS. Moreover, the 2.17 Mb region between qHRC-C6-1 and qHRC-C6-2 on ChrA6, with 48.27% PVE in total, was considered the prominent QTL region, and the genes in the region were retrieved and annotated. This prominent QTL region will be further finely mapped to identify candidate genes related to the NR content. The high-density TKS genetic map and mapping results obtained in this work will provide valuable information for future candidate gene identification, map-based gene cloning, and MAS in TKS.

## Materials and methods

### Plant material, DNA extraction and phenotype evaluation

TKS 166 plants with a low rubber content (LRC) (2.09%) and X51 plants with a high rubber content (HRC) (9.21%) were obtained from a TKS population, collected from Zhaosu, Xinjiang Province, China, in 2016 and preserved in the TKS germplasm nursery of the Rubber Research Institute, the Chinese Academy of Tropical Agricultural Sciences (CATAS) in Haikou (China). The NR content for 166 and X51 was further quantified and identified according to the method of Yang et al. [34] in the greenhouse and open field of the TKS germplasm nursery (Haikou, China) in 2019, respectively. The two TKS lines were established and preserved by tissue culture to overcome self-incompatibility. The mapping population consisting of 127 F_1_ progeny was generated by crossing lines 166 and X51. The 127 F_1_ progeny and two parental lines were grown in an open field of the TKS germplasm nursery (Rubber Research Institute, Haikou, China). After 2 months of cultivation, approximately 1 g of young healthy leaves from each plant in the F_1_ mapping population was collected, cleaned, and stored at -80 °C for DNA extraction. Genomic DNA was extracted with a Super Plant Genomic DNA Kit (TIANGEN BIOTECH, Beijing, China), quantified with a NanoDrop 1000 spectrophotometer (NanoDrop, Wilmington, DE, USA), and further evaluated with 1.0% agarose gel electrophoresis.

After 6 months of cultivation, a total of 1 g fresh roots for each plant in the F_1_ mapping population were collected, dried and ground into powder for determination of the NR content according to the method of Yang et al. [34]. Three repetitions were carried out for each plant, and the final NR content was the average value of three tests.

### Library construction and sequencing

After checking the quality of genomic DNA, the DNA samples were randomly fragmented into 200–500 bp fragments through sonication, purified, and terminally repaired, and 3’ A and a sequencing linker were added. Then, the sequencing libraries were constructed by fragment size selection using agarose gel electrophoresis and PCR amplification [29]. Finally, the library was quality inspected and sequenced on the Illumina HiSeq platform at Biomarker Technology Co. Ltd. (Beijing, China). The low-quality reads were filtered out from the raw reads by deleting the reads with adapters, filtering reads with an N content higher than 10%, and removing the reads containing more than 50% bases (Q ≤ 10). High-quality raw reads were obtained and sorted for each sample according to barcode sequences.

### SNP identification and genotyping

Burrows-Wheeler Aligner software was used to map the clean reads onto the TKS reference genome sequences [43], and the sequencing depth, genome coverage ratio, and read distribution for each sample were calculated and analyzed. Samtools and bcftools (https://github.com/samtools/bcftools) were used to mark and eliminate duplicated reads, and base recalibration was used to detect potential SNPs, which were called with default parameters on each sample.

SNPs were further filtered, and those with different genotypes in both parents, quality scores ≥ 30, and MQ scores ≥ 30 were retained. All polymorphic SNPs were genotyped based on genotype uniformity in the parental and progeny SNP loci. To ensure the genetic map’s quality, SNPs with a sequencing depth less than 3 were removed.

### Genetic map construction

A genotype matrix from the 127 F_1_ progenies was generated, and the genetic distances were calculated using MSTMap (http://mstmap.org/). After screening, the SNPs were divided into bin markers that were filtered with lengths greater than 1000. The genetic map was constructed using the bin marker data generated by MG2C (http://mg2c.iask.in/mg2c_v2.0). The ALLMAPS program was used to construct the chromosomes [44].

### QTL mapping and gene information retrieval in QTL regions

The QTL loci related to the NR content were identified using the R/QTL analysis method [45]. The recombination rate and LOD values were calculated using the maximum likelihood estimation method. The bin markers with LOD values ≥ 4.0 were selected as QTLs.

Information on candidate genes located in the QTL region was retrieved according to the TKS genomic sequences and gene prediction information (GWHBCHF00000000; GWH: http://bigd.big.ac.cn/gwh/). Functional annotation of those genes was conducted using BlastX (version 2.10.1+) in the NCBI nonredundant protein sequences (nr) database. The statistical enrichment and classification of those genes in KEGG pathways were conducted using KOBAS software (www.kegg.jp/kegg/kegg1.html) [46].

## Supplementary information

**Additional file 1: Fig. S1. **Distribution of SNP quality in resequenced lines. R1 and R2 represent the two parents. R3-R129 represent the individuals of the F_1_ population. **Fig. S2. **Distribution of SNP mutation types in resequenced lines. R1 and R2 represent the two parents. R3-R129 represent the individuals of the F_1_ population. **Fig. S3. **KEGG classification of genes in the region between qHRC-C6-1 and qHRC-C6-2 (www.kegg.jp/kegg/kegg1.html).

**Additional file 2: Table S1. **Results of the resequencing of the parents and 127 F_1_ population. **Table S2. **The position of all the bin markers mapped on the map ChrA0/LG00: sequences assemble that is not integrated into a specific chromosome. **Table S3.** The genotype of all the markers mapped on the map. b: Recessive homozygous genotype (166)  h: Dominant heterozygous genotype  a: Dominant homozygous genotype. **Table S4.** Summary of genes in the region between qHRC-C6-1 and qHRC-C6-2. **Table S4.** Summary of genes in the region between qHRC-C6-1 and qHRC-C6-2. **Table S6.** The genetic effects of  QTLs on NR content in the F_1_ population. Note: + represents the genotype of parent X51; - represents the genotype of parent 166. **Table S7.** Genotypes of six major QTLs in F_1_ population.

## Data Availability

The WGR raw sequence data and variation data of the F_1_ mapping population generated and analyzed in this study are available in the Genome Sequence Archive (https://ngdc.cncb.ac.cn/gsa.) and the Genome Variation Map (GVM) (https://bigd.big.ac.cn/gvm) [47] in National Genomics Data Center, Beijing Institute of Genomics, Chinese Academy of Sciences and China National Center for Bioinformation [48], under accession number CRA009020 and GVM000440 respectively. The TKS genome information was deposited in the Genome Warehouse database under the accession number GWHBCHF00000000 (GWH: http://bigd.big.ac.cn/gwh/).

## Abbreviations

AFLP: Amplified fragment length polymorphism
COS: Conserved ortholog set
CTAB: Cetyltrimethyl ammonium bromide
DXS: 1-Deoxy-d-xylulose-5-phosphate synthase
GBS: Genotyping-by-sequencing
GGPS: Geranylgeranyl diphosphate synthase
Hb: Hevea brasiliensis
HRC: High rubber content
IPP: Isopentenyl pyrophosphate
KOBAS: KEGG Orthology-Based Annotation System
LG: Linkage group
LOD: Logarithm of odds
LRC: Low rubber content
MAS: Marker-assisted selection
MEP: 2-C-methyl-D-erythritol-4-phosphate
MVA: Mevalonic acid
NGS: Next-generation sequencing
nr: Nonredundant
NR: Natural rubber
PVE: Phenotypic variance value
QTL: Quantitative trait locus
SNP: Single nucleotide polymorphism
SSR: Simple sequence repeat
TF: Transcription factor
TKS: *Taraxacum kok-saghyz* Rodin
WGR: Whole-genome resequencing
