# Supplementary material for: Construction of the first high-density SNP genetic map and identification of QTLs for the natural rubber content in Taraxacum kok-saghyz Rodin
Source: BMC Genomics. 2023 Jan 10;24:13. doi: 10.1186/s12864-022-09105-3 (PMC9830913; doi:10.1186/s12864-022-09105-3)
Supplement: Supplementary file 1 — Additional file 1: Fig. S1. Distribution of SNP quality in resequenced lines. R1 and R2 represent the two parents. R3-R129 represent the individuals of the F1 population. Fig. S2. Distribution of SNP mutation types in resequenced lines. R1 and R2 represent the two parents. R3-R129 represent the individuals of the F1 population. Fig. S3. KEGG classification of genes in the region between qHRC-C6-1 and qHRC-C6-2 (www.kegg.jp/kegg/kegg1.html). [file 12864_2022_9105_MOESM1_ESM.docx]

**Construction of the first high-density SNP genetic map and identification of QTLs for the natural rubber content in *Taraxacum kok-saghyz* Rodin**

Yushuang Yang^1^, Bi Qin^1^, Qiuhui Chen^1^, Qiuhai Nie^2^, Jichuan Zhang^3^, Liqun Zhang^3^, Shizhong Liu^1,*^

(^1^Rubber Research Institute, Chinese Academy of Tropical Agricultural Science, Haikou 571101;

^2^Beijing Linglong Dandelion Technology and Development Ltd, Beijing, 101102;

^3^College of Materials and Engineering, Beijing University of Chemical Technology, Beijing 100029)


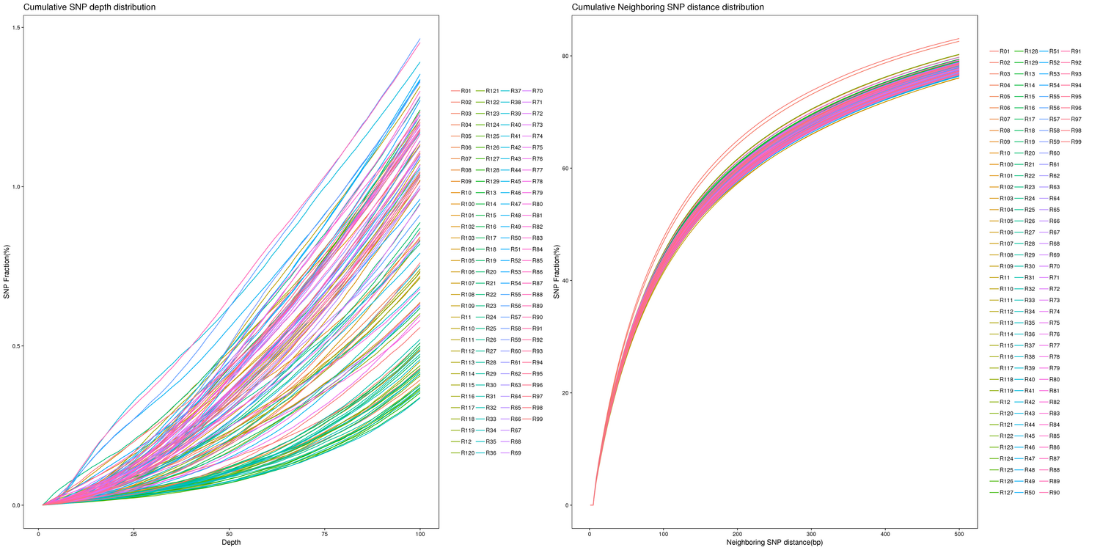


**Fig. S1. Distribution of SNP quality in resequenced lines.** R1 and R2 represent the two parents. R3-R129 represent the individuals of the F_1_ population.


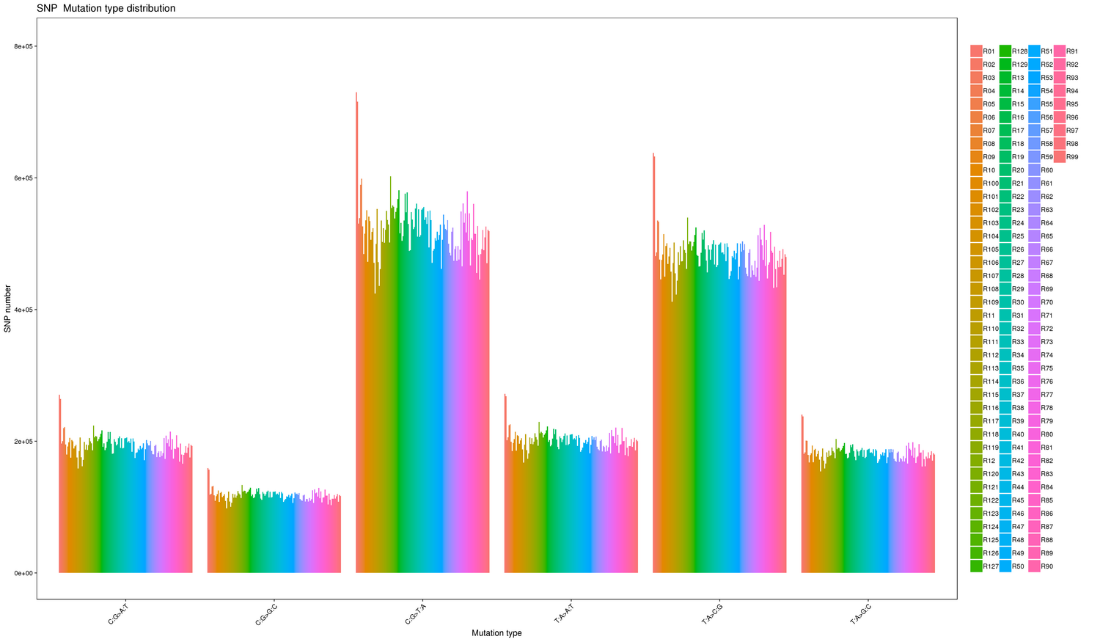


**Fig. S2. Distribution of SNP mutation types in resequenced lines.** R1 and R2 represent the two parents. R3-R129 represent the individuals of the F_1_ population.


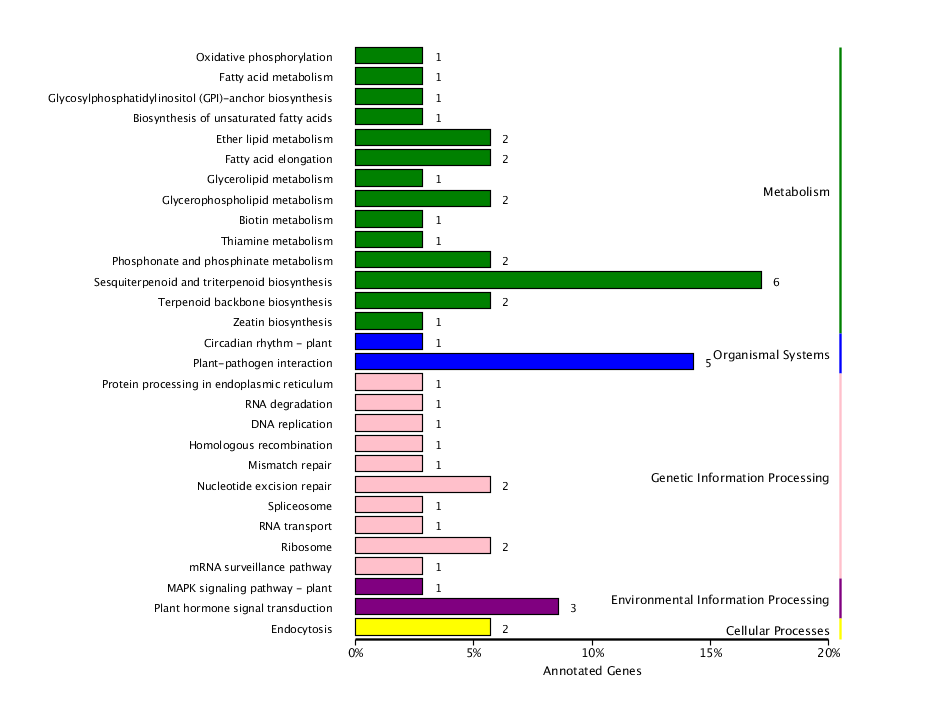


**Fig. S3. KEGG classification of genes in the region between qHRC-C6-1 and qHRC-C6-2 （www.kegg.jp/kegg/kegg1.html）.**
